# Draft genome sequence of *Monascus ruber* strain FM39-7

**DOI:** 10.1128/mra.00805-23

**Published:** 2023-12-15

**Authors:** Lei Yang, Yongfeng Jing, Zhijun Cheng, Ke Huang, Xianhai Yang, Dong Xiang, LudwigSimaneka Omuwa Hilde, Huilin Zhang, Yong Liu

**Affiliations:** 1 China Tobacco Hunan Industrial Co. Ltd., Changsha, China; 2 School of Biological & Chemical Engineering, Zhejiang University of Science & Technology, Hangzhou, China; University of Guelph, Guelph, Ontario, Canada

**Keywords:** draft genome, *Monascus ruber*, edible fungi

## Abstract

In China, certain *Monascus ruber* strains are traditionally used as edible fungi. We sequenced the genome of *M. ruber* FM39-7 strain, an isolate from fermented rice. The genome is 25.89 Mb with a G + C content of 48.86%, containing 8485 annotated genes.

## ANNOUNCEMENT

*Monascus* species, including *Monascus ruber*, *Monascus fumeus*, *Monascus purpureus*, and *Monascus pilosus*, have been traditionally used as edible fungi in China due to their ability to produce red and yellow pigments (1), γ-aminobutyric acid (2), lovastatin (3), and their association with the fungal toxin citrinin (4). These strains can be found in various sources, including soil, fermented crops, and beverages. In this study, we present the draft genome of *M. ruber* strain FM39-7, an isolate from a fermented rice sample (at 30° 18’ N, 120° 8’ E). The pure FM39-7 culture was obtained by single spore isolation, and the sequencing mycelium sample was collected after 7-day incubation on Potato Dextrose Agar at 35°C.

The genomic DNA of *M. ruber* FM39-7 was extracted using the sodium dodecyl sulfate (SDS) method (5), and its quality was assessed by agarose gel electrophoresis and quantified using Qubit. The DNA samples were broken into target fragments by Covaris g-TUBE, purified and selected by AMPure PB magnetic beads, and then used for library construction. The genome was sequenced using an Illumina HiSeq 2500 by PE150 strategy with three DNA libraries constructed with insert size of 350 bp, 2.0 kb, and 6.0 kb, respectively. Quality control and filtering of reads were conducted to reach Q30 (%) at of 88.25%, 88.91%, and 90.03% in clean data, respectively. The assembly was constructed using SOAPdenovo2 (6, 7) . Predictions of open reading frame (ORF), *t*RNA genes, *r*RNA genes, and *s*RNAs were performed using GeneMarkS (version 4.17) by self-training using Hidden Markov model, tRNAscan-SE (8), rRNAmmer 1.2 (9), and BLAST against the Rfam (10) database, respectively. Repeat sequences were identified using RepeatMasker (version 4.0.5) (11), and tandem repeats were analyzed using Tandem Repeat Finder (version 4.07b) (12). Default parameters were used except where otherwise noted.

The sequencing process generated clean data of 5,158 Mb, 4,930 Mb, and 3,525 Mb, corresponding to insert sizes of 350 bp (read length 125:125 bp), 2,000 bp (read length 150:150 bp), and 6,000 bp (read length 150:150 bp), respectively. A total of 32 scaffolds and 255 contigs were constructed, with a GC content of 48.86% and an N50 value of 312,939 bp. The genome annotation was performed based on databases of GO, KEGG, COG, KOG, eggNOG, NR, TCDB, Swiss-Prot, and TrEMBL, revealing a total of 8,485 genes. Additionally, 325 secreted proteins were predicted using SignalP 4.1, neural networks, Hidden Markov models, and the TMHMM tool.The enzymes associated with carbohydrate-based metabolites *Monascus* pigments, lovastatin, and citrinin also were annotated by Carbohydrate-Active enZYmes Database which find 18 carbohydrate-binding modules, 7 carbohydrate esterases, 115 glycoside hydrolases, 63 glycosyltransferases, 2 polysaccharide lyases, and 25 auxiliary activities.

## Data Availability

The data of this Whole Genome Shotgun project has been deposited at DDBJ/ENA/GenBank under the accession JAVJAL000000000. The version described in this paper is version JAVJAL010000000. Additionally, the data can be found in the CNGB Sequence Archive (CNSA) (13) of the China National GeneBank DataBase (CNGBdb) (14) with the accession number CNP0004716.
